# Poisoning with the Berries of Atropa Belladona, or Deadly Nightshade

**Published:** 1846-12

**Authors:** 


*Poisoning with the berries of Atropa Belladona, or deadly Nightshade.*—In ruined and desolate places, in the skirts of woods, among the offal of a garden, there grows a "wicked weed" which our ancestors called dwale. Its very name is portentous of misery and woe. Unlike some of those plants which conceal their venom under a fair aspect, or disguise it by a fragrant odour, this is fœtid in its leaves, and repulsive in its flowers, which are dull pale chocolate coloured bells, with a lurid yellow bottom. There is not a point of beauty about the thing till it bears its fruit, but then it becomes only too attractive. No cattle will touch it; not a fly or grub finds a resting-place or a pasture among its leaves, and it may even be said to be shunned by its own species, for it usually grows year after year singly, in the same place, without a companion near it.

The root of this plant is a thick tap, deeply plunged in the earth. Its stems grow about two or three feet high, and produce from their forks the solitary nodding blossoms. The leaves are dull green, and pretty constantly in pairs on one side of the stem, as if they had been half torn from their sockets, and misplaced in the setting. They are unequal in size, have a soft almost greasy feel, and are in form not unlike an egg cut through lengthwise, but sharper. In each of the bell-shaped flowers are five stamens adhering to the sides, and a roundish two-celled ovary, with one style, many ovules, and a kidney-shaped hairy stigma, with a pair of blubber lips. The border of the corrolla is regularly cut into five divisions. As soon as the flower drops off, it is succeeded by a green ball, filled with small seeds; this, as it swells, becomes distended with deep purple juice, till at last it grows into a fruit, succulent, sweetish to the taste, tempting to the eye, and not unlike a black cherry.

In Latin it is called belladona, a word signifying *fair lady*, and first applied by the Italians. The learned Bodæus a Stapel, says it obtained this name, because people took a potion prepared from it in order to procure pleasant dreams, of which handsome women invariably formed a part. But its true history is that a cosmetic was once prepared from it. Distilled dwale-water was said to remove freckles, and to render the complexion fair and white. But it has long been forgotten, the gentle sex having discovered that "le meilleur de tous les cosmétiques est une vie sobre et régulière ; il conserve la santé, et la fraicheur qui en est l'expression." We now call it the deadly nightshade, but the Germans term it *wolf's cherry*, and *devil's berry*, and thereby point out how much it is to be shunned.

These names reveal its nature. It is a fatal poison in every part—berries, leaves, stem, and root; and the first are a fruitful source of fatal accidents. Half a berry is said to have caused death. Hence the fruit has been occasionally used as an intentional poison. The old chroniclers tell of a legion of Danes having been feasted by the Scotch, who finished them off by a mess of dwale from which they never woke ; a German case is recorded of death having been caused by crushing the berries in wine ; an old woman is said to have killed a person by a potion prepared by boiling the flower-buds in water.

Accident, however, is more commonly the cause of injury from these berries. They are gathered in wild places by ignorant people, who think no ill of a fruit so fair. A French writer mentions an instance of 150 soldiers having been poisoned near Dresden ; and less extensive instances occur frequently, of which we have at this time an example, in the case of a man now lying in prison to take his trial for poisoning persons in London, by selling the berries for tarts.

Of these cases, frenzy is a symptom ; persons eating them become maniacs in a few hours ; and this quality is characteristic of the whole plant, for when Shakespeare called the deadly nightshade the "insane root," he only expressed poetically its well-known properties. "Even the dried root produces insanity," are the very words of Haller. Hence the names of the old herbalists, who call it the mortiferous, somniferous, or furious Solanum.

It is, however, certain, that some constitutions are able to resist this poison much better than others. It seems, doubtful, indeed, whether any effect at all is produced by small quantities upon some people. Haller lays it down as a rule, that three or four may be eaten without danger; and he asserts that he has seen a larger number swallowed with impunity by a medical student of Cologne, named Simon. A Danish gardener once employed near London, is said to have habitually swallowed a berry when annoyed by the impertinencies of his companions; and a case is quoted by Dr. Christison of a French lad who ate a pound of the berries before going to bed, and nevertheless recovered, although he was not subjected to medical treatment till the next morning. These facts give some colour to the assertion of the man now awaiting his trial, that he did not know the berries to be poisonous, because he had eaten them himself.

But how deplorable it is that the population of a city like London should be so profoundly ignorant as not to know these berries when offered for sale. We hear of no policeman stopping Hillard's trade; indeed it was at first supposed that they were sloes that he was selling; even the reporters in the police-courts seem to have known no better. Would it not be as well if as much botany were introduced into our national schools as would prevent such fatal consequences as these? A very little instruction would render such mistakes impossible.—*Gardeners' Chronicle, from Dub. Med. Press.*

